# Relative tree cover does not indicate a lagged Holocene forest response to monsoon rainfall

**DOI:** 10.1038/s41467-022-33958-7

**Published:** 2022-10-24

**Authors:** Ying Cheng, Yue Han, Hongyan Liu

**Affiliations:** 1 School of Geography and Tourism, Shaanxi Normal University, Xi’an, 710119 China; 2 College of Urban and Environmental Sciences and MOE Laboratory for Earth Surface Processes, Peking University, Beijing, 100871 China

**Keywords:** Palaeoecology, Palaeoclimate

**arising from** J. Cheng et al. *Nature Communications* 10.1038/s41467-021-22087-2 (2021)

Recently, Cheng J. et al.^1^ cited and simulated the relative percentage tree cover to interpret the ~3000–4000 years lag between tree cover and East Asian Summer Monsoon (EASM) rainfall. They concluded that vegetation feedback has caused a lagged ecosystem response to EASM rainfall during the Holocene (11.7–0 ka). Here, we question the feasibility of using the relative percentage tree cover to measure vegetation feedback to climate. First, the land cover in northern China includes forests, grasslands, and bare land^2^. Cheng J. et al.^1^ did not consider the role of bare land in climate feedback models. Absolute land cover, including forest, grassland, and bare land can accurately reveal feedback to climate^3^. Second, the biome reconstructions they cited represent changes in vegetation type only, whereas the relationship between vegetation type and vegetation cover is altered by many other factors^3–5^. Third, the paper they cited^6^ averaged the vegetation types on a millennium scale with an interval of 1000 years, so the view that vegetation has a ~3000–4000 years lag in EASM rainfall is not credible as the lag can be enlarged by data resolution. Therefore, absolute vegetation cover, not relative cover, is a prerequisite for studying ecosystem feedback.

Our previous work^3^ was the first to reconstruct the absolute vegetation cover in northern China based on pollen concentrations in two well-dated sediment cores. Using a random forest method, the vegetation cover at Dali Lake in the forest-steppe transition in northern China was determined for the period from 19,000 cal. yr BP to the present with a resolution of approximately 200 years. Han et al.^3^ showed that tree cover peaked during the early Holocene and it has gradually declined since the middle Holocene. Pollen percentages are widely used in vegetation reconstruction, but they are challenging to analyze because they can be similar in composition, despite being produced by very different flora^7,8^. This means they represent the relative fractions of vegetation type and not the absolute vegetation cover (Fig. 1A, B). Han et al.^3^ suggested that pollen concentration data are suitable for the reconstruction of absolute vegetation cover, particularly in arid and semi-arid regions. Dali Lake represents a typical lake in the semi-arid area of northern China. The random forest model showed that the area had a high tree cover during the early Holocene, which suggests that tree cover was a timely response to Holocene monsoon rainfall and there was no time lag at this specific location. Therefore, the data at Dali Lake challenge the conclusion that tree cover has a ~3000–4000 years lag to EASM rainfall.

**Fig. 1:**
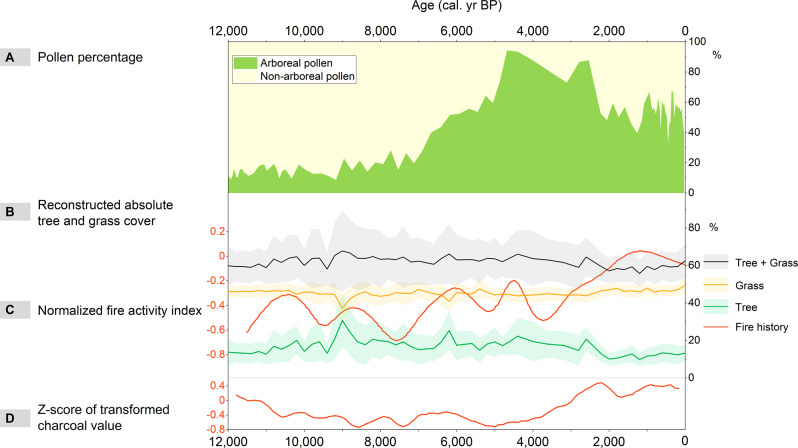
Trends in pollen percentage, absolute vegetation cover and fire history during the Holocene at Dali Lake, a typical lake in the semi-arid area of northern China. Changes in the percentages of arboreal and non-arboreal pollen (**A**)^3^. Reconstructed absolute tree and grass cover (**B**)^3^, with gray, yellow, and green shaded areas indicating the standard deviation of 1000 random forest model results for total cover, grass cover, and tree cover, respectively. Normalized fire activity index in the northern region of eastern monsoonal China (**C**)^10^. Z-score of transformed charcoal value showing fire activity trend in the temperate steppe of northern China (**D**)^11^.

Moreover, Cheng J. et al.^1^ used −17 °C as the threshold for tree and grass transition, but this could be an incorrect citation from Bonan et al.^9^. In the original text by Bonan et al.^9^, they showed that both temperate deciduous broadleaved trees and C_3_ grasses have a tolerance of −17 °C for the coldest month for their survival, but this temperature is not the favored threshold for the shift from grasses to trees. Actually, the trend in absolute vegetation cover was mainly driven by summer temperature, annual precipitation, and fire incidents, which is in line with the vegetation-climate relationships at Dali Lake^3^. That is, higher monsoon rainfall could increase the competitiveness of trees, while increased fire could increase the competitiveness of grasses as grasses are mostly annual and perennial, and they renew faster than trees after a fire. Between 10,000 and 8000 cal. yr BP, monsoon rainfall peaked and there were relatively few fires, which led to a significant increase in absolute tree cover. Since 6500 cal. yr BP, monsoon rainfall decreased and fire increased, resulting in stronger competition by grasses, which has led to an increased grass cover and reduced tree cover^3^ (Fig. 1B).

The impact of secondary disturbances on vegetation dynamics requires careful consideration, particularly the impact of fires on vegetation cover in semi-arid areas of China, even though vegetation growth is strongly constrained by rainfall in this region. Both the normalized fire index in the northern region of eastern monsoonal China^10^ and the charcoal value in the temperate steppe of northern China^11^ show a clear antiphase relationship with the absolute forest cover of the Dali Lake region (Fig. 1B, C, D). However, Cheng J. et al.^1^ did not discuss fire incidence on vegetation evolution in northern China. Fire has occurred frequently through the Holocene^10,12^ and it plays an important role in vegetation dynamics based on observational evidence^13,14^. Particularly, fire is considered as a triggering disturbance that can reduce a forest’s resilience to drought under a drying climate during the mid to late Holocene. For Dali Lake, fire and drying climate has co-driven the evolution of vegetation cover since 6500 cal. yr BP^3^. Moreover, in northern China, data from Daihai Lake and Hulun Nuur Lake also suggest that fire has accelerated the decline of forest cover and the transition from forest to grass during the Holocene^13,14^. Unfortunately, Cheng J. et al.^1^ did not discuss the effect of fire when citing and interpreting the observation data, as scale-dependent fires could make the transition between forest and grassland and their interactions variable.

In summary, we believe there are three flaws in the data interpretation of Cheng J. et al.^1^. (1) They contradict the observed evolution of absolute tree cover at Dali Lake in northern China, which is representative of the marginal area of EASM. Relative percentage tree cover cannot accurately reflect a forest’s response and feedback to past climate change. (2) Both temperate deciduous broadleaved trees and C_3_ grasses have a tolerance of −17 °C for the coldest month for their survival. Thus, −17 °C is not a correct threshold for the shift from grasses to trees. (3) They contradict the ecological theories of secondary disturbance on vegetation dynamics. Fire and other secondary disturbances may be crucial to the transition between forest and grassland. Interpretations of vegetation feedback might be biased if these important factors are not fully considered. Based on the evidence, their conclusion that vegetation feedback causes lagged ecosystem response to EASM rainfall during the Holocene could be problematic.

## Data Availability

The data used herein are available at 10.18170/DVN/V9JN52.
